# Ferulic acid ameliorates lipopolysaccharide-induced tracheal injury via cGMP/PKGII signaling pathway

**DOI:** 10.1186/s12931-021-01897-4

**Published:** 2021-12-04

**Authors:** Xiaoyong Xie, Tong Yu, Yapeng Hou, Aixin Han, Yan Ding, Hongguang Nie, Yong Cui

**Affiliations:** 1 Department of Anesthesiology, the First Hospital of China Medical University, Shenyang, 110001 China; 2 Department of Stem Cells and Regenerative Medicine, College of Basic Medical Science, China Medical University, Shenyang, 110122 China

**Keywords:** Ferulic acid, Tracheal injury, Epithelial sodium channel, cGMP/PKGII signaling pathway

## Abstract

**Background:**

Tracheal injury is a common clinical condition that still lacks an effective therapy at present. Stimulation of epithelial sodium channel (ENaC) increases Na^+^ transport, which is a driving force to keep tracheal mucosa free edema fluid during tracheal injury. Ferulic acid (FA) has been proved to be effective in many respiratory diseases through exerting anti-oxidant, anti-inflammatory, and anti-thrombotic effects. However, these studies rarely involve the level of ion transport, especially ENaC.

**Methods:**

C57BL/J male mice were treated intraperitoneally with normal saline or FA (100 mg/kg) 12 h before, and 12 h after intratracheal administration of lipopolysaccharide (LPS, 5 mg/kg), respectively. The effects of FA on tracheal injury were not only assessed through HE staining, immunofluorescence assay, and protein/mRNA expressions of ENaC located on tracheas, but also evaluated by the function of ENaC in mouse tracheal epithelial cells (MTECs). Besides, to explore the detailed mechanism about FA involved in LPS-induced tracheal injury, the content of cyclic guanosine monophosphate (cGMP) was measured, and Rp-cGMP (cGMP inhibitor) or cGMP-dependent protein kinase II (PKGII)-siRNA (siPKGII) were applied in primary MTECs, respectively.

**Results:**

Histological examination results demonstrated that tracheal injury was obviously attenuated by pretreatment of FA. Meanwhile, FA could reverse LPS-induced reduction of both protein/mRNA expressions and ENaC activity. ELISA assay verified cGMP content was increased by FA, and administration of Rp-cGMP or transfection of siPKGII could reverse the FA up-regulated ENaC protein expression in MTECs.

**Conclusions:**

Ferulic acid can attenuate LPS-induced tracheal injury through up-regulation of ENaC at least partially via the cGMP/PKGII pathway, which may provide a promising new direction for preventive and therapeutic strategy in tracheal injury.

**Supplementary Information:**

The online version contains supplementary material available at 10.1186/s12931-021-01897-4.

## Introduction

Normal tracheal lumen is lined with polarized columnar cells with cilia on the top, constituting the predominant structural barrier to pathogens and a variety of environmental agents [1], which represents a pivotal site for the innate immunity and host defense [2]. Mouse tracheal epithelial cells (MTECs) cultured in air–liquid mode have been well-established cell models to investigate respiratory diseases, the morphological and physiological characteristics of which were consistent with those of respiratory epithelial cells in vivo [3]. Epithelial sodium channel (ENaC) is a heteromultimeric protein mainly composed of α, β, and γ subunits, among which α subunit is necessary to form functional ENaC, while the β and γ subunits can promote the channel’s activity [4, 5]. Previous studies have reported that alveolar edema could hardly be cleared in mice lacking either α-, β- or γ-ENaC gene, which proves the important role of ENaC in fluid homeostasis [6–8]. Patients with tracheal injury are associated with reductions in ENaC protein expressions [9, 10]. In addition, it has been reported that abnormalities in tracheal cilia structure and function lead to a decrease of ENaC activity, which contributes to the occurrence and development of chronic respiratory diseases [11].

Lipopolysaccharide (LPS), a major biologically active component of the gram-negative bacterial cell wall, has been widely used to induce a tracheal injury model, that is similar with pathological features to trachea lesions in humans by triggering excessive inflammatory mediator. Various respiratory epithelial cells, such as club cells, ciliated cells, and type II alveolar cells, were damaged after LPS challenge, evidenced by the reduced expression of relative cell markers [12]. Many lines of evidence indicate that exposed to LPS stimulation can cause inflammatory response and other tracheal lesions, leading to the occurrance of respiratory disorders [13, 14].

Ferulic acid (FA) (4-hydroxy-3-methoxycinnamic acid) is a natural phytochemical, which is widely found in traditional Chinese medicine such as Angelica, Chuanqi, and Ferula. FA has low toxicity and possesses many physiological functions (anti-inflammatory, antioxidant, antimicrobial activity, anticancer, and antidiabetic effect) [15], which has been proved to be effective in many disease models such as lung injury, diabetes, epilepsy, and hepatotoxicity [16, 17]. Nevertheless, the research on the mechanism of FA in respiratory diseases is still limited, most of which stays at the level of inflammatory factors, free radicals and inflammatory cell infiltrations, and rarely involves the level of ion transport [18, 19]. Although FA can attenuate LPS-induced lung injury through anti-inflammatory and anti-oxidant activities, there are no detailed studies about the regulation of FA on ENaC, which may also be relevant with the anti-inflammatory effects in LPS induced tracheal injury. Our previous studies showed that cyclic guanosine monophosphate (cGMP)-dependent protein kinase II (PKGII), a downstream of cGMP, is one of the activators of ENaC [20, 21]. In addition, it has also been found that FA induces proliferation and differentiation of osteoblasts through cGMP/PKGII/ENaC signaling pathways [22]. We speculate that FA can regulate ENaC through the cGMP/PKGII pathway in MTECs, so as to alleviate tracheal injury induced by LPS.

## Materials and methods

### LPS-induced tracheal injury model in mice

All animal experiments were carried out on 8-week-old male C57BL/J mice (18–22 g) of SPF grade, purchased from Huafukang Biotechnology (Beijing, China). All experimental protocols relating to mice were performed according to the guidelines and regulations of Animal Care and Use Ethics Committee, and were approved by China Medical University (No. CMU2020088). Mice were randomly divided into six groups: Control, LPS, FA (Sigma, Saint Louis, MO, USA), FA + LPS, LPS + Rp-cGMP, and FA + LPS + Rp-cGMP group [19]. The mice were intratracheally instilled with 5 mg/kg LPS (LPS, FA + LPS LPS + Rp-cGMP, and FA + LPS + Rp-cGMP group) and 5 μg/kg Rp-cGMP (LPS + Rp-cGMP and FA + LPS + Rp-cGMP group), respectively. Meanwhile, mice in FA, FA + LPS, and FA + LPS + Rp-cGMP group were injected intraperitoneally with FA (100 mg/kg) 12 h before and 12 h after LPS treatment (0.9% NaCl in Control group). [23, 24]. After LPS administration for 12 h, the mice were sacrificed and trachea tissues were collected for the following experiments.

### HE staining and immunofluorescence assay

The trachea tissues were fixed in 4% paraformaldehyde, embedded in paraffin and then sectioned at 5 μm thickness. The sections were stained with hematoxylin and eosin (HE) kit (Beyotime, Shanghai, China) to determine the pathological changes in ciliated tracheal epithelium. Then four representative views (original objective × 400) of HE staining from each group were selected, and the average height of tracheal epithelium as well as number of epithelial cells per area was measured to evaluate the changes of ciliated epithelial cells in trachea. The immunofluorescence was done after the trachea tissues were fixed in 4% paraformaldehyde, dehydrated in 30% sucrose, embedded in OCT, and cut into 8 μm slices. The cell membrane was permeabilized by Triton-100 (0.1%) and visualized by incubating with γ-ENaC (1:200 dilution, 4 ℃, overnight) and FITC labeled goat-anti-rabbit (1:100 dilution, room temperature, 90 min in a dark humidifying box). The nucleus was stained by DAPI.

### Cell culture

Healthy C57BL/J male mice were anaesthetized by diazepam (17.5 mg kg^−1^) followed 6 min later by ketamine (450 mg kg^−1^) intraperitoneally. Removed tracheas were digested with 3 ml 0.1% protease XIV, 0.01% DNA enzyme, and 1% FBS in DMEM for 24 h at 4 °C. Cell precipitation was resuspended with 1 ml DMEM (containing 5% FBS) and centrifuged twice at 2000 rpm/min. Subsequently, MTECs were resuspended again and seeded onto 6.5-mm diameter mouse tail collagen I pre-coated transwell inserts (Corning Costar, Lowell, MA, USA) at a density of 3.0 × 10^5^ cells/cm^2^, while 0.5 ml complete medium was added to lower part of the transwell compartments. The complete medium consists of 1:1 mixture of 3T3 conditioned medium (containing 4 mM Glutamine, 4500 mg/l Glucose, 10% FBS, 100 U/ml penicillin, 100 μg/ml streptomycin) and Ham’s F-12 medium (containing 1 mM Glutamine), appended with 10 μg/ml insulin (Sigma, Saint Louis, MO, USA), 1 μM hydrocortisone (Sigma, Saint Louis, MO, USA), 250 nM dexamethasone (Sigma, Saint Louis, MO, USA), 3.75 μg/ml bovine endothelial cell growth supplement (Cell Applications, San Diego, CA, USA), 25 ng/ml epidermal growth factor (Sigma, Saint Louis, MO, USA), 10 ng/ml cholera toxin (Macgene, Beijing, China), 30 nM triiodothyronine (Sigma, Saint Louis, MO, USA), 5 μg/ml iron saturated transferring (Gibco, New York, NY, USA) and 30 μg/ml bovine pituitary extract (SceinCell, San Diego, CA, USA). 4 days after culture, upper layer culture medium in transwell was removed to establish air–liquid interface and the medium was changed every two days. After 12 days, MTECs with a trans-epithelial electrical resistance > 1000 Ω/cm^2^, were applied to Ussing chamber experiments (Additional file 1: Fig. S1).

### CCK-8 cell viability assay

Cell viability was measured using a CCK-8 assay (Biosharp, Guangzhou, China) according to the manufacturer’s protocol. MTECs were plated at a density of 1 × 10^4^ cells/well in 96-well plates. After cultured overnight, the cells were treated with different dosage of FA (0, 25, 50, 100, 200, 400, 800 μM, respectively) for 24 h. After culture, CCK-8 reagent (10 μl) was added to each well containing 100 μl culture medium and the plate was incubated at 37 °C for 1 h. Cell viability was evaluated by absorbance measurements at 450 nm.

### Ussing chamber assay

Measurements of trans-epithelial electrical resistance and short-circuit current (*Isc*) were performed as previously described [25]. MTEC monolayers were installed in Ussing chambers (Physiologic Instruments, San Diego, CA, USA), perfused with (mM, pH 7.4) 120 NaCl, 25 NaHCO_3_, 3.3 KH_2_PO_4_, 0.83 K_2_HPO_4_, 1.2 CaCl_2_, 1.2 MgCl_2_, and 10 HEPES, supplemented with either 10 mannitol (apical compartment) or 10 glucose (basolateral compartment). The osmolality of each solution was between 290 and 300 mOsm/kg, and both sides of the bath solutions were bubbled with 95% O_2_ and 5% CO_2_ continuously at 37 °C. MTEC monolayers were short circuited to 0 mV, and a 10 mV pulse of 1 s was given every 10 s to monitor trans-epithelial electrical resistance. When the *Isc* and resistance were stable, 100 μM ENaC specific inhibitor amiloride was added to apical bath solution. While the *Isc* was stable again, the data was analyzed with the Acquire and Analyze program version 2.3.

### Inhibition of cGMP or PKGII knockdown in MTECs

The MTECs were treated with the inhibitor of cGMP (Rp-cGMP) (Sigma, Saint Louis, MO, USA) (10 μM) for 12 h, or transfected with PKGII-siRNA (siPKGII) (GenePharma, Shanghai, China) (200 nM) for 72 h according to the manufacturer’s protocol, respectively. The specific siRNA primers are forward (5′-CUG UUG GAA G UG GAA UAC UA-3′), and reverse (5′-UAG UAU UCC ACU UCC AAG AG-3′). The protein expression levels of PKGII and ENaC subunits were analyzed.

### Western blot assay

The cell lysates were separated by SDS-PAGE (10% polyacrylamide gels) and transferred onto PVDF membrane (Invitrogen, Waltham, MA, USA). After blocking with 5% nonfat dried milk in Tris-buffered saline containing 0.05% Tween 20, the membranes were incubated with primary antibodies α-ENaC (1:2000, PA1-920A, Thermo Fisher, Waltham, MA, USA), γ-ENaC (1:2000, ab3468, Abcam, Cambridge, MA, USA), PKGII (1:2000, sc-393126, Santa Cruz Biotechnology, Santa Cruz, CA, USA), and β-actin (1:1000, sc-47778, Santa Cruz Biotechnology, Santa Cruz, CA, USA) overnight at 4 °C. The membranes were washed three times with TBST for 10 min each time and then reacted with horseradish peroxidase-conjugated secondary antibody, goat-anti-rabbit or goat-anti-mouse secondary antibody (1:5000, ZSGB-BIO, Beijing, China) at room temperature for 1 h. The protein bands were visualized using ECL kit on a Tanon-5200 chemiluminescence detection system (Tanon, Shanghai, China), and the intensity of each specific band was quantified with Image J program.

### Quantitative real-time polymerase chain reaction

Trizol reagent (Invitrogen, Waltham, MA, USA) was used to extract total RNA from trachea tissues and MTECs, and concentration and purity of RNA were detected by NanoDrop 2000C spectrophotometer (Thermo, Wilmington, DE, USA). RNA samples with D260/280 and D260/230 both in 1.8–2.0 were synthesized into cDNA by the instructions of the PrimeScript RT reagent kit (TaKaRa, Kusatsu, Shiga, Japan). Quantitative real-time polymerase chain reaction (qRT-PCR) was then applied using SYBR Premix Ex Taq II (TaKaRa, Kusatsu, Shiga, Japan) in the ABI 7500 qRT-PCR System with the following primers: α-ENaC forward (5′-AGG GCT GAG CCT AGA GCT AGA GA-3′) and reverse (5′-TTC CTC CCG GAC TGT TTG AC-3′), β-ENaC forward (5′-GGG ACC AAA ACC ACC TTA GCT GCC ATC AC-3′) and reverse (5′-TGC AGT ACC ACA CTA GCA GC-3′), γ-ENaC forward (5′-CAG CCG TGA CCC TTC AGT TC-3′) and reverse (5′-CCT TAA TGG TCG GCG CCT GG-3′), and GAPDH forward (5′-AGA AGG CTG GGG CTC ATT TG-3′) and reverse (5′-AGG GGC CAT CCA CAG TCT TC-3′). Reverse transcription reaction conditions were 37 °C for 15 min, and 85 °C for 5 s. Polymerase chain reactions comprised of pre-denaturation at 95 °C for 30 s, 40 cycles of denaturation at 52 °C for 30 s, and annealing at 60 °C for 34 s. The experimental results were analyzed by 2^−Δ(ΔCT)^ relative quantitative method.

### ELISA assay

According to the manufacturer’s instructions, the cGMP levels of tissue homogenate was determined using ELISA Kit (Jiang Lai, Shanghai, China). The optical density (OD) values were measured at the wavelength of 450 nm using a microplate reader (Tecan, Mannedorf, Switzerland), and the concentrations of cGMP were calculated according to the standard curves. The results were presented as pmol/mg protein.

### Statistical analyses

The experimental results were expressed by mean ± standard error. The data were tested by Levene and Shapiro–Wilk test for normality and homogeneity of variance, and the differences were compared by Student’s paired-sample. Otherwise, one-way analysis of variance (ANOVA) test as well as Bonferroni's test (for unpaired data) was used for mean comparison. When the data did not pass the normality or homoscedasticity test, we used a non-parametric t-test (Mann–Whitney U test). All experimental data are statistically analyzed by Origin 8.0, and *P* < 0.05 is considered to be statistically significant.

## Results

### FA attenuated LPS-induced tracheal injury

**Fig. 1:**
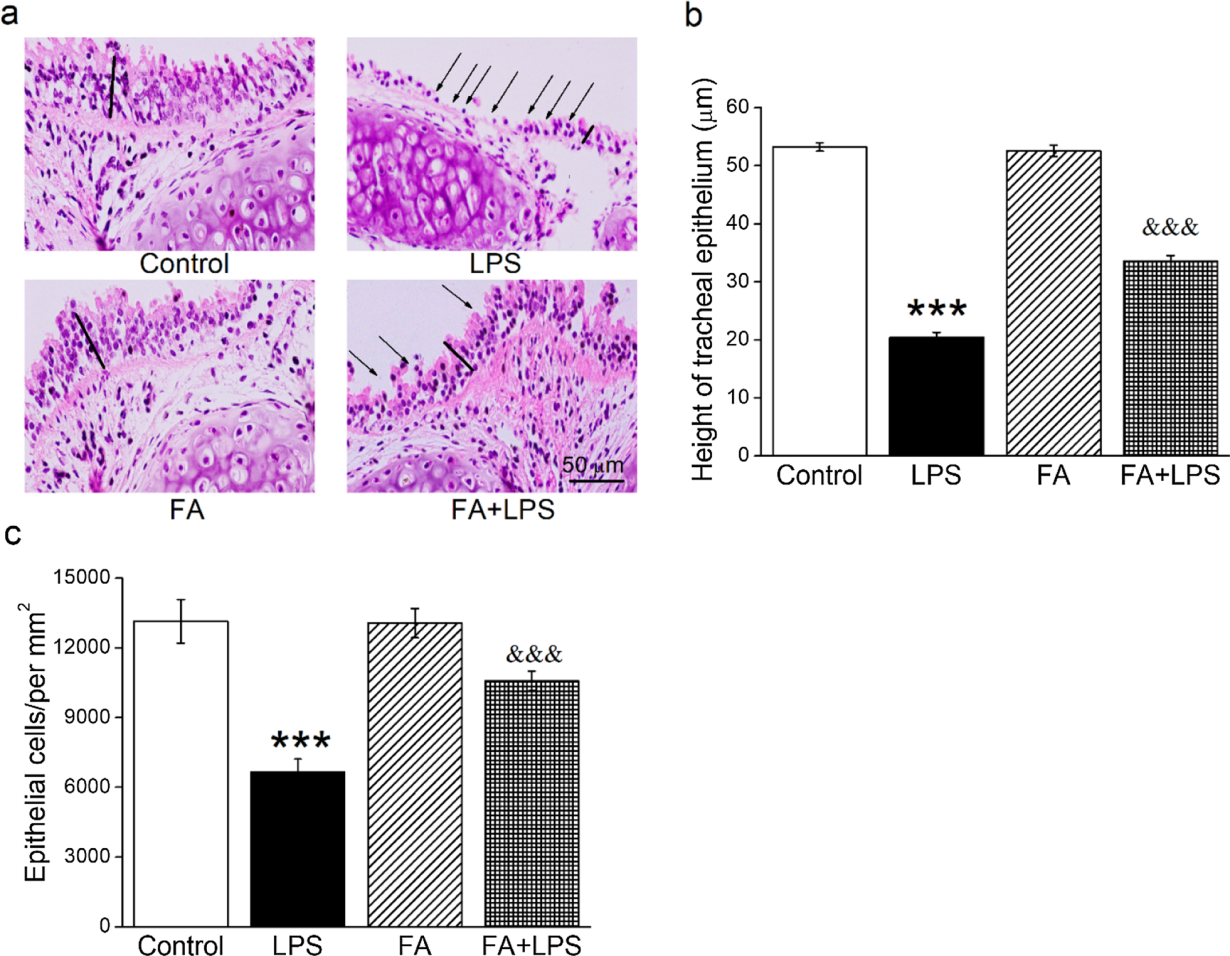
The pathological changes of mice with tracheal injury were improved by FA. **A** C57BL/J male mice were treated intraperitoneally with normal saline or FA (100 mg/kg) 12 h before and 12 h after intratracheal administration of LPS (5 mg/kg), and then extracted tracheas were applied to hematoxylin and eosin (HE) staining (original objective × 400). The black arrow indicates the abnormal ciliated columnar epithelia, and the line segment represents the height of trachea epithelium. **B** Five points were randomly selected to measure the height of tracheal epithelium in each group. **C** Number of tracheal epithelial cells per area. ***P < 0.001, compared with Control group, ^&&&^*P* < 0.001, compared with LPS group, n = 5

The intratracheal surface is lined with pseudostratified ciliated columnar epithelium, and we applied HE staining to evaluate the pathological morphology of tracheal injury in mice. As shown in Fig. 1A, after exposure to LPS, a large number of ciliated columnar cells were lost in the trachea and the pseudostratified structure was destroyed, even converted into a monolayer columnar structure (arrow position). After the administration of FA, the impaired pseudostratified structure of tracheal epithelium in LPS-induced mice was partially recovered. In order to quantitatively compare the protective effect of FA on tracheal injury, the height of 5 sites of tracheal epithelium was randomly measured (denoted by line segments) and then the average of them was calculated. Our results showed that LPS significantly decreased both the height of tracheal epithelium and number of epithelial cells per area (Fig. 1B, C, P < 0.001, compared with Control group), which was reversed by the co-administration of FA (*P* < 0.001, compared with LPS group).

### Protein/mRNA expression levels of ENaC were up-regulated after FA administration

**Fig. 2:**
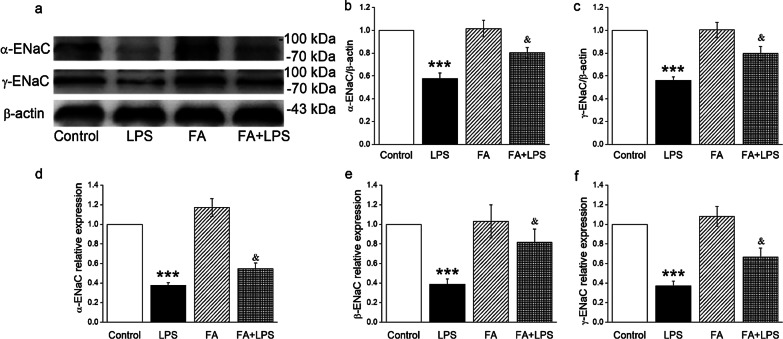
Effects of FA on the proteins and transcriptional expression level of ENaC in the trachea of mice. **A** C57BL/J male mice were treated intraperitoneally with normal saline or FA (100 mg/kg) 12 h before and 12 h after intratracheal administration of LPS (5 mg/kg), subsequently the proteins were extracted and analyzed by Western blot. The typical bands of α- and γ-ENaC protein expression and β-actin were used as internal controls. **B** and **C** Graphical representation of data obtained from Western blot assays for α- and γ-ENaC subunits, which were quantified using gray analysis (α-ENaC/β-actin and γ-ENaC/β-actin). **D**–**F** mRNA samples were isolated from mouse models of tracheal injury. Relative levels of mRNA were calculated as α- or β- or γ-ENaC/GAPDH ratios. ***P < 0.001, compared with Control group, ^&^*P* < 0.05, compared with LPS group, n = 4–5

ENaC functions as a limiting factor for Na^+^ uptake, and is responsible for salt-water transport in trachea. To clarify the ENaC regulation of FA during tracheal injury, we determined the protein and mRNA expressions of ENaC, which were measured by Western blot and qRT-PCR, respectively. We found that α- and γ-ENaC protein expression levels were down-regulated in LPS-treated trachea tissues (Fig. 2B, C, P < 0.001, compared with Control group), which were enhanced after FA treatment (*P* < 0.05, compared with LPS group). We didn’t detect the β-ENaC expression for the lack of suitable antibody for the Western blot assay.

Due to the results that FA could increase the expression of ENaC proteins, we speculate that the transcription levels of ENaC are also enhanced after FA administration. Next we tried qRT-PCR to determine the mRNA expression levels of the three subunits of ENaC and as expected, the decreased mRNA expressions of α, β, and γ-ENaC were remarkably reversed by the FA treatment (Fig. 2D–F, P < 0.05, compared with LPS group).

### FA enhanced amiloride-sensitive short-circuit currents in MTECs

**Fig. 3:**
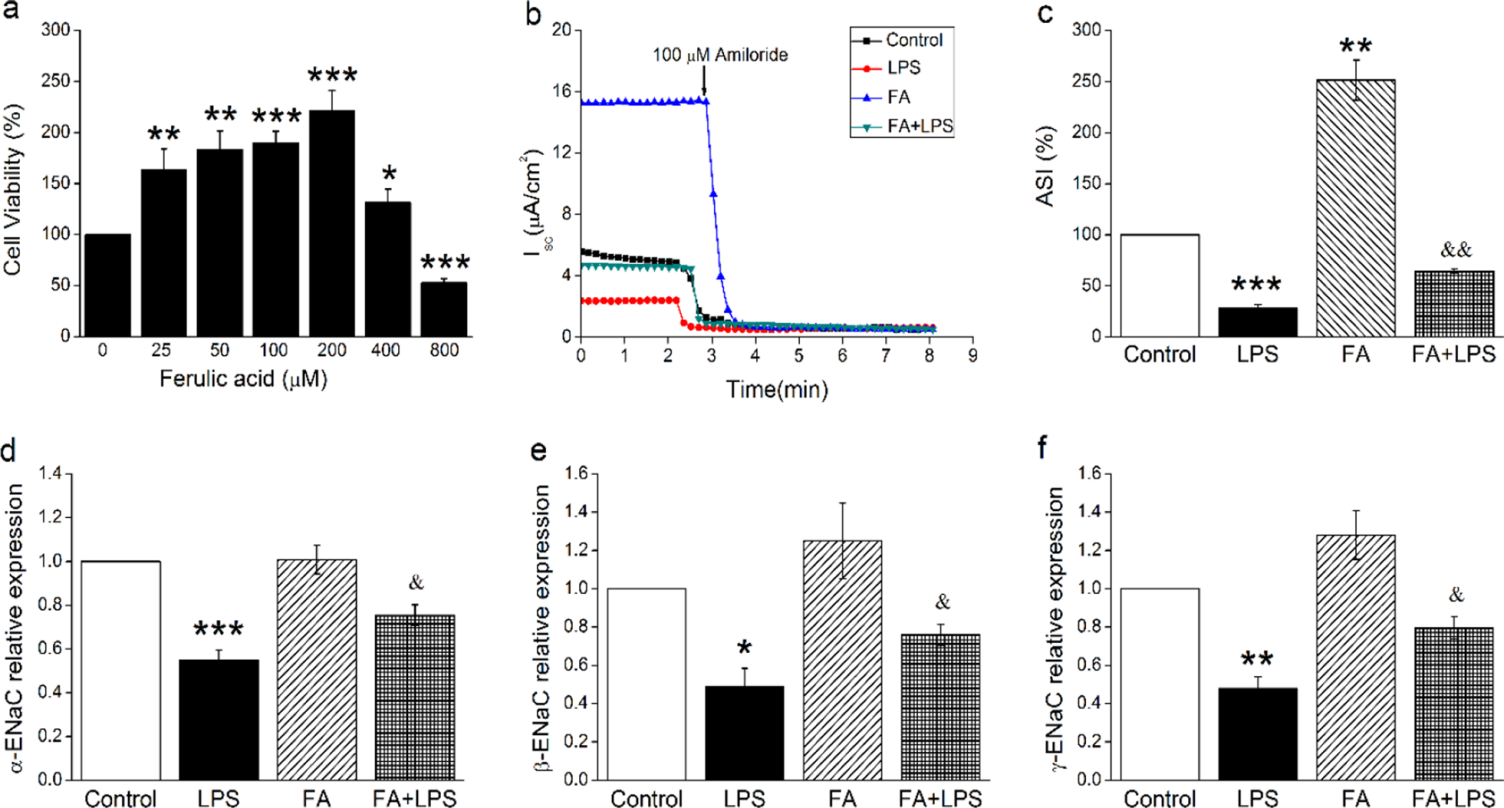
*Isc* level and mRNA expression of ENaC is enhanced by FA in MTEC monolayers. **A** The MTECs were incubated with different doses of FA (0, 25, 50, 100, 200, 400, 800 μM) for 24 h, and cell counting kit-8 (CCK-8) assay was performed to examine cell viability. In addition, the FA concentration of 0 μM was used as 100%. *P < 0.05, **P < 0.01, ***P < 0.001, compared with 0 μM group, n = 5–7. **B** *Isc* traced after MTEC monolayers were treated with LPS for 12 h and FA for 24 h, then amiloride (100 μM) was applied. **C** Statistic ASI in MTEC monolayers. ASI was defined as the difference between the total current and the amiloride-resistant current and the initial ASI was set as 100%. **D**–**F** Relative level of ENaC mRNAs were calculated as α-, β- or γ-ENaC/GAPDH ratios. *P < 0.05, **P < 0.01, ***P < 0.001, compared with Control group; ^&^*P* < 0.05, ^&&^*P* < 0.01, compared with LPS group, n = 3

Then we explored the ENaC-related effects and mechanisms of FA on LPS-damaged tracheal epithelial cells at the cellular level. In order to determine the optimal FA dosage that significantly influenced cell viability, the CCK-8 assay was carried out in MTECs. As illustrated in Fig. 3A, 25–200 μM FA could increase the viability of MTECs when compared with 0 μM group, which was concentration-dependent and statistically significant (*P* < 0.001–0.05). Whereas the concentration of FA reached 400–800 μM, the proliferation ability of MTECs began to decline, resulting in toxic effects. Accordingly, we chose 200 μM as the concentration for the subsequent experiments [26].

The amiloride specifically inhibits ENaC activities, while being a poor inhibitor of other channels and transporters at lower concentrations, thus amiloride-sensitive *Isc* (ASI) is used to measure ENaC activity in MTECs [27]. We defined ASI as the difference between the total current and the amiloride-resistant current, and set the initial ASI as 100%. The experiment results from Ussing chamber assay indicated that LPS induced a reduction of ASI in MTECs, which could be reversed by 200 μM FA (Fig. 3B, C, P < 0.01, compared with LPS group).

We also repeatedly detected the effects of FA on ENaC at the transcription level in MTECs, which was consistent with those in tracheal tissues (Fig. 3D–F). Overall, FA could not only increase the ENaC expression, but also its function in LPS-induced tracheal injury.

### The cGMP content was enhanced after FA treatment

**Fig. 4:**
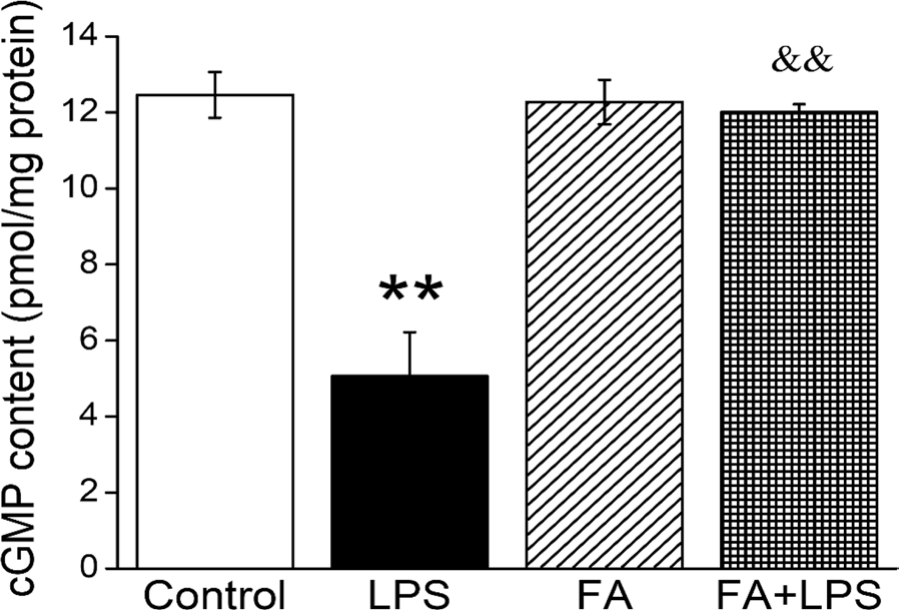
FA increased cGMP levels. C57BL/J male mice were treated intraperitoneally with normal saline or FA (100 mg/kg) 12 h before and 12 h after intratracheal administration of LPS (5 mg/kg). cGMP content was detected by ELISA kit. **P < 0.01, compared with Control group; ^&&^*P* < 0.01, compared with LPS group, n = 4–5

cGMP, one of intracellular second messengers, is responsible for regulating sodium channels in rat trachea, and we hypothesized that FA may also enhance the expressions and activities of ENaC in mouse tracheal epithelium by cGMP [28]. The cGMP content was measured by ELISA kit and as shown in Fig. 4, cGMP was decreased obviously in LPS group (*P* < 0.01, compared with Control group), which was abrogated after FA administration (200 μM, *P* < 0.01, compared with LPS group).

### FA-induced ENaC protein expression elevation was dependent on cGMP

**Fig. 5:**
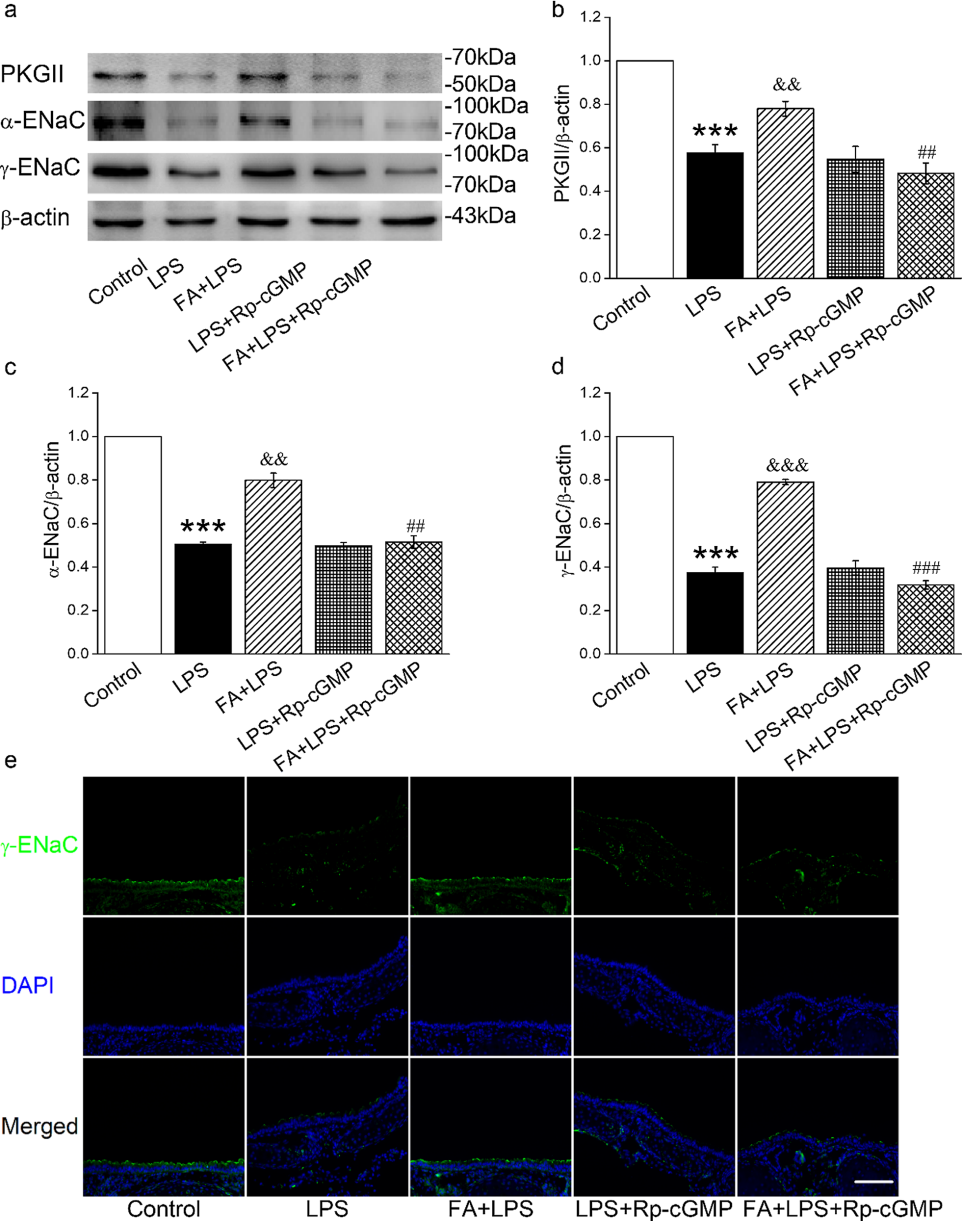
The FA increased ENaC expression was dependent on cGMP. MTECs were treated with Rp-cGMP (cGMP inhibitor, 10 μM) in the presence of FA (200 μM) and LPS (10 ng/ml) for 12 h. After incubation, the cells were harvested. PKGII and α/γ-ENaC protein expressions in the cell lysates were detected by Western blot assay. **A** Representative Western blot bands of PKGII and α/γ-ENaC protein expression in LPS-induced MTECs treated with Rp-cGMP. **B**–**D** Graphical representation of data obtained from Western blot assays for PKGII and α/γ-ENaC. Bands were quantified using gray analysis (PKGII/β-actin, α-ENaC/β-actin, and γ-ENaC/β-actin). ***P < 0.001, compared with Control group; ^&&^*P* < 0.01, ^&&&^*P* < 0.001, compared with LPS group; ^##^*P* < 0.01, ^###^*P* < 0.001, compared with FA + LPS group, n = 4. **E** Immunofluorescence images of γ-ENaC in mouse tracheal tissue. scale bar = 100 μm

To investigate whether cGMP is involved in the FA enhanced protein expressions of ENaC in tracheal epithelium, we applied Rp-cGMP (cGMP inhibitor, 10 μM) and compared the ENaC expressions by Western blot in primary MTECs. As shown in Fig. 5A, B, the protein level of PKGII, a downstream of cGMP, was decreased significantly in the LPS group (*P* < 0.001, compared with Control group). When co-administration of FA, the inhibitory effect of LPS on PKGII were reversed (*P* < 0.01, compared with LPS group). After cells were incubated with Rp-cGMP in the presence of FA for 24 h and LPS for 12 h, the protein expression level of PKGII was markedly decreased (*P* < 0.01, compared to FA + LPS group). Both the expression levels of α- and γ-ENaC were consistent with the trend of PKGII (Fig. 5C, D), whereas there was no significant difference in PKGII and ENaC protein expressions between LPS + Rp-cGMP and LPS + FA + Rp-cGMP group (*P* > 0.05). To test the localized upregulation of ENaC expression with FA in vivo, we performed immunofluorescence assay in mouse trachea. The results showed that γ-ENaC was mainly expressed in the epithelial cells, and Rp-cGMP significantly abrogated the effect of FA on LPS induced ENaC reduction (Fig. 5E).

### PKGII gene knockdown reversed the upregulation effect of FA on α/γ-ENaC in MTECs

**Fig. 6:**
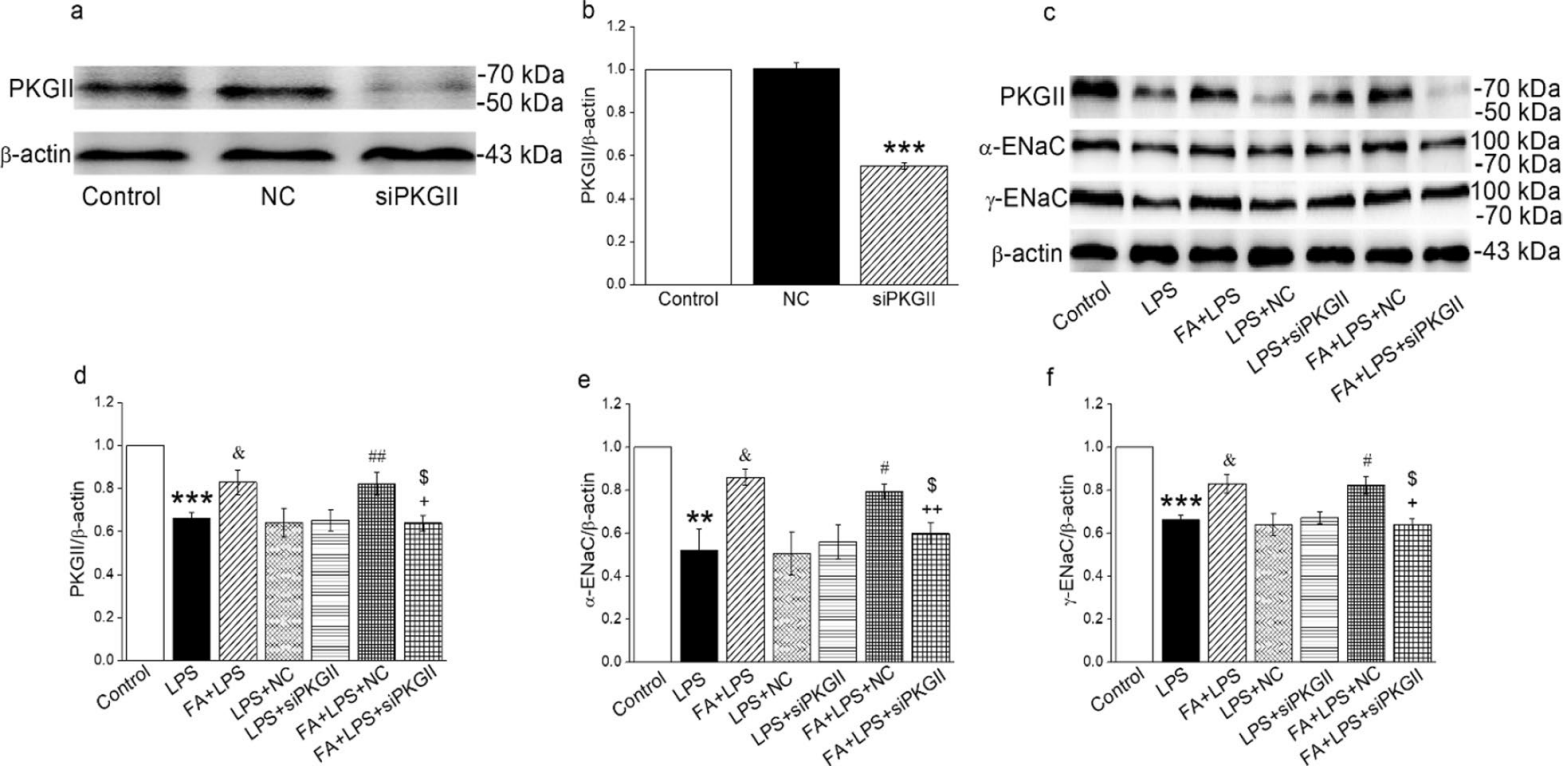
PKGII gene knockdown reversed the upregulation effect of FA on the protein expression of α/γ-ENaC in MTECs. **A**–**B** Representative Western blot bands and statistic data for PKGII knockdown. ***P < 0.001, compared with negative control (NC), n = 4. **C** Representative Western blot bands of PKGII and α/γ-ENaC protein expression in LPS-treated MTECs transfected with siPKGII. **D**–**F** Graphical representation of data obtained from Western blot assays for PKGII and α/γ-ENaC. Bands were quantified using gray analysis (PKGII/β-actin, α-ENaC/β-actin, and γ-ENaC/β-actin). **P < 0.01, ***P < 0.001, compared with Control group; ^&^*P* < 0.05, compared with LPS group; ^#^*P* < 0.05, ^##^*P* < 0.01, compared with LPS + negative control (NC), ^+^*P* < 0.05, ^++^*P* < 0.01, compared with FA + LPS group, ^$^*P* < 0.05, compared with FA + LPS + negative control (NC), n = 4

**Fig. 7:**
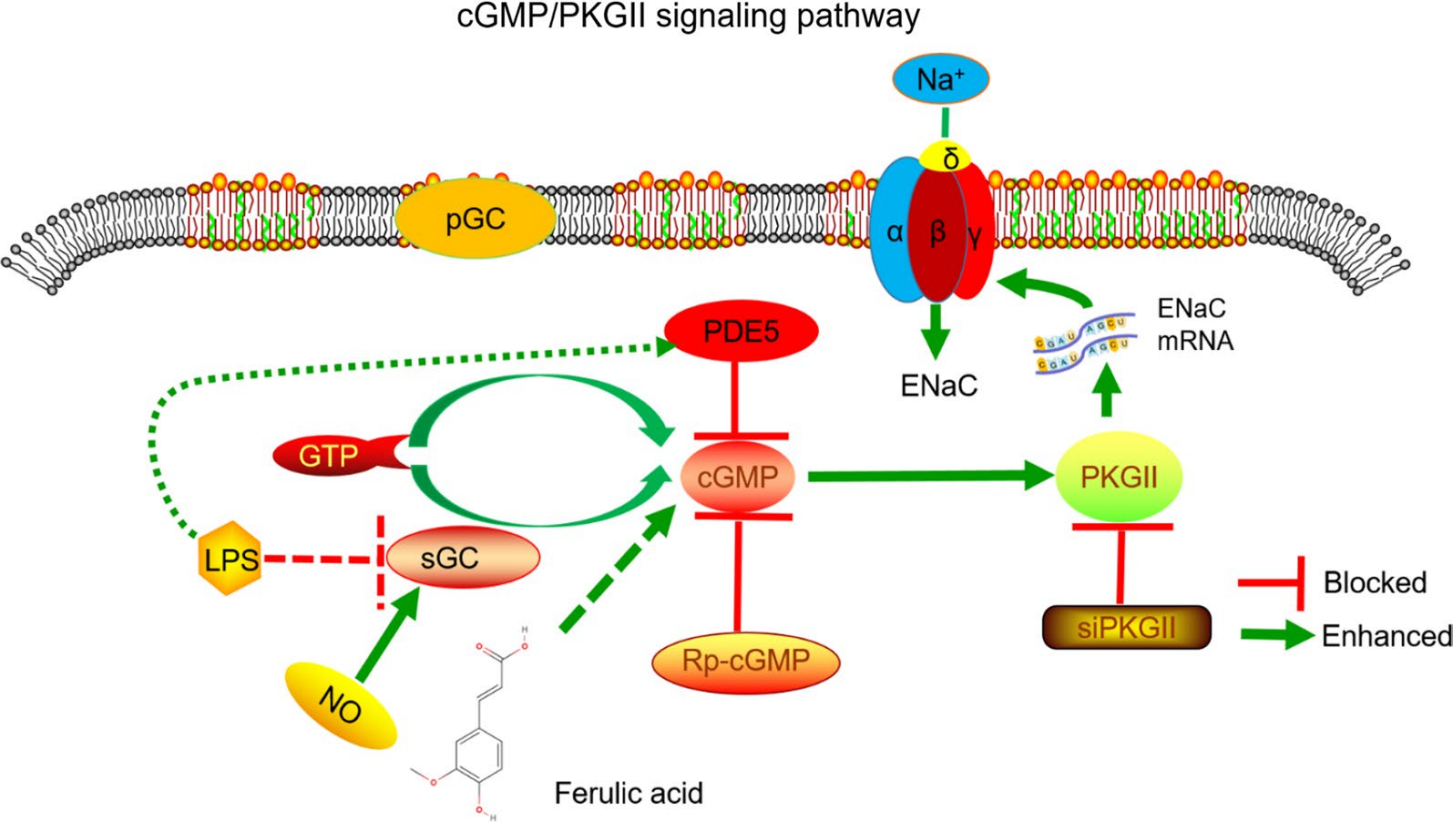
A schematic diagram for FA to enhance the activities and expressions of ENaC in LPS-induced MTECs via cGMP/PKGII signaling pathway. Under normal circumstances, guanosine triphosphate (GTP) is translated into cyclic guanosine monophosphate (cGMP) in the existence of membrane-bound guanylate cyclase (pGC) and free guanylyl cyclase (sGC). LPS is able to induce a desensitization of the sGC/cGMP-dependent pathway by decreasing protein expression levels of sGC-β1and increasing phosphodiesterase 5 (PDE5) activities, hence cGMP is degraded drastically. FA can interdict the effect of LPS by increasing the expression of cGMP as well as downstream-cGMP-dependent protein kinase II (PKGII), which enhances the mRNA/protein expression and function of ENaC in MTECs, and can be reversed when MTECs are treated with Rp-cGMP (a special inhibitor for cGMP) or transfected with a small interference RNA targeted PKGII (siPKGII)

To further test whether FA enhanced the expressions of ENaC on tracheal epithelium through cGMP/PKGII signaling pathway, we transfected MTECs with siPKGII to find out its role in the regulation of ENaC. We first identified the siRNA transfection efficiency by the Western blot analysis (Fig. 6A, B). Similar with the results of the above cGMP inhibitor, both the PKGII and α/γ-ENaC protein expression levels were inhibited in cells transfected with siPKGII for 72 h, which were reversed by FA (Fig. 6D–F). There was still no significant change between LPS + siPKGII and LPS + FA + siPKGII group (*P* > 0.05). These results supported that FA may improve tracheal injury by upregulating ENaC expression via the cGMP/PKGII pathway (Fig. 7).

## Discussion

Tracheal epithelium is not only a mechanical barrier to external stimuli and microbes, but also actively involved in innate and acquired immune responses during tracheal inflammation [29]. Constantly exposed to viruses, bacteria, or dust particles, the tracheal barrier structure will be destroyed, and the immune cells which then be activated by secreting a variety of cytokines and chemokines, thereby promoting the occurrence of tracheal injury [30]. Thus, tracheal epithelial cells have been speculated to be a key regulator of tracheal pathophysiology in various tracheal injury-related respiratory diseases, such as allergic asthma, chronic obstructive pulmonary disease, and cystic fibrosis [31].

LPS is derived from the outer membrane of gram-negative bacteria, which can induce the features of acute inflammation in the tracheal epithelium, facilitating extensive tissue damage [32], and LPS-induced tracheal injury is a popular model that is conforming to medical research requirements [12, 33]. However, despite several important advances in tracheal injury treatment during the last few decades, the specific preventive/therapeutic role and mechanisms of tracheal injury are still undetermined. Therefore, discovering new drugs and new therapeutic targets remains an urgent priority.

FA is extracted from natural plants and has various biological activities, including anti-oxidant and anti-inflammation effects [34]. In the past few years, FA has been found to rescue LPS-induced neurotoxicity via modulation of the toll-like receptor 4 in the mouse hippocampus [35]. Studies also proved that FA alleviated LPS-induced acute respiratory distress syndrome through its anti-inflammatory, and improved survival in mice [18]. Moreover, sodium ferulae and oxymatrine could regulate IL-1β and INF-γ, so as to relieve lung injury induced by LPS [36, 37]. However, whether FA has similar protective impacts on LPS-induced mouse tracheal injury, still requires further study.

ENaC is mainly composed of α, β and γ subunits in mammalian respiratory epithelial cells, which belongs to the voltage independent ion channels, and participates in maintaining appropriate salt and water balance by reabsorbing Na^+^ at the apical membrane, thereby creating an osmotic gradient that facilitates the reabsorption of fluid. As an important channel for epithelial fluid and electrolyte transport, ENaC located on tracheal epithelial cells plays a key role in the hyperresponsiveness diseases and asthmatic attacking [10]. Meanwhile, ENaC is closely associated with inflammatory mediators, which participate in both lung and tracheal injury [38, 39]. Previous studies demonstrated that allergic tracheal inflammation is closely associated with the transcription level decrease of ENaC [38, 40]. Therefore, ENaC is expected to participate in regulating the imbalance of fluid transport in trachea, which is a characteristic of tracheal injury.

Considering the slow effects of traditional Chinese medicine, the pretreatment pattern of drug administration is used to explore the mechanisms of FA in our in vivo studies. The results showed that FA could improve the pathological changes of tracheal injury, maintaining the pseudostratified ciliated columnar structure of tracheal epithelial cells. Moreover, FA could reverse the effect of LPS on the expression levels of ENaC in trachea as expected, which were measured by qRT-PCR, Western blot, and immunofluorescence assay, respectively. The morphology and physiological characteristics of the MTECs cultured in air–liquid mode were consistent with those of the respiratory endothelial cells in vivo, which is a good cell model for the study of respiratory diseases, and has been used in toxicology, respiratory infection, ion migration, cell carcinogenesis, etc. [41]. In our experiment, the effect of FA on ENaC activity was measured by Ussing chamber assay and after LPS-induced MTEC monolayers were treated with FA for 24 h, the ASI level was increased, which was probably related to the enhanced expression of ENaC [42].

Previous studies have proved that ENaC is regulated by multiple signaling pathways. For example, insulin can alleviate LPS-induced acute lung injury by upregulating ENaC through PI3K/Akt/SGK1 signaling pathway [43]. Evidence from our group has shown that PKGII, a downstream molecule of cGMP, is an ENaC activator [20, 44]. Moreover, it has been reported that FA can up-regulate ENaC in mouse osteoblasts through cGMP/PKGII signaling pathway, thus promoting osteoblastic differentiation [44]. Therefore, we investigated whether FA also affected ENaC in tracheal epithelium through cGMP/PKGII signaling pathway. Our results proved that FA treatment abrogated the reduction of cGMP after LPS treatment and after the administration of Rp-cGMP or siPKGII, the FA-enhanced ENaC protein expression was inhibited in MTECs, supporting that FA may improve tracheal injury by upregulating ENaC expression via the cGMP/PKGII pathway. On the consideration that FA and other Chinese medicine monomers are attracting more and more attention of medical researchers these years, this study will provide new theoretical support and broad application prospects for FA in the prevention and treatment of edematous respiratory diseases.

## Conclusions

FA can attenuate LPS-induced tracheal injury through up-regulation of ENaC at least partially *via* the cGMP/PKGII pathway, which may provide a promising new direction for preventive and therapeutic strategy in tracheal injury.

## Supplementary Information

**Additional file 1: Figure S1.** A schematic diagram for the experiment setup. The transmembrane MTEC monolayer was acquired on the air-liquid interface culture mode from day 4-12.

## Data Availability

The datasets used and analyzed during the current study are available from the corresponding author on reasonable request.

## Abbreviations

ASI: Amiloride-sensitive short-circuit current
CCK-8: Cell counting kit-8
cGMP: Cyclic guanosine monophosphate
ELISA: Enzyme-linked immunosorbent assay
ENaC: Epithelial sodium channels
FA: Ferulic acid
GC: Guanosyl cyclase
GTP: Guanosine triphosphate
Isc: Short-circuit currents
LPS: Lipopolysaccharide
MTECs: Mouse tracheal epithelial cells
PKGII: CGMP-dependent protein kinase II
qRT-PCR: Quantitative real-time polymerase chain reaction
