# Supplementary material for: Ferulic acid ameliorates lipopolysaccharide-induced tracheal injury via cGMP/PKGII signaling pathway
Source: Respir Res. 2021 Dec 4;22:308. doi: 10.1186/s12931-021-01897-4 (PMC8642995; doi:10.1186/s12931-021-01897-4)
Supplement: Supplementary file 1 — Additional file 1: Figure S1. A schematic diagram for the experiment setup. The transmembrane MTEC monolayer was acquired on the air-liquid interface culture mode from day 4-12. [file 12931_2021_1897_MOESM1_ESM.docx]

**Ferulic acid ameliorates lipopolysaccharide-induced tracheal injury *via*** **cGMP/PKGII signaling pathway**

**Xiaoyong Xie^1,2#^, Tong Yu^2#^, Yapeng Hou^2^, Aixin Han^2^, Yan Ding^2^, Hongguang Nie^2^**^*^**, Yong Cui^1*^**

^1^Department of Anesthesiology, the First Hospital of China Medical University, Shenyang, 110001, China

^2^Department of Stem Cells and Regenerative Medicine, College of Basic Medical Science, China Medical University, Shenyang, 110122, China.

# These authors contribute equally to this work.

*Correspondence: Yong Cui, ycui@cmu.edu.cn; Hongguang Nie, [hgnie@cmu.edu.cn](mailto:hgnie@cmu.edu.cn)


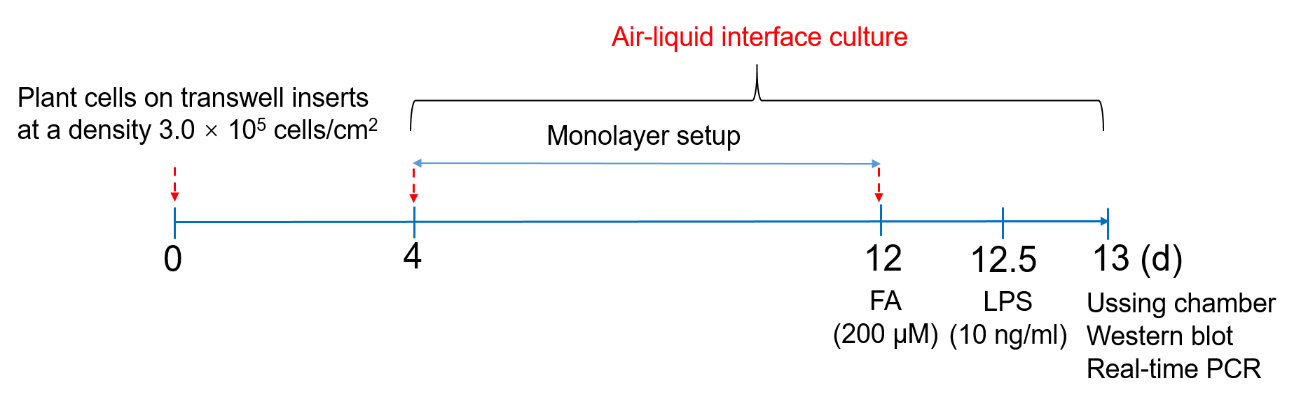


**Supplementary Fig. 1.** A schematic diagram for the experiment setup. The transmembrane MTEC monolayer was acquired on the air-liquid interface culture mode from day 4-12.
